# Mechanisms for exporting large-sized cargoes from the endoplasmic reticulum

**DOI:** 10.1007/s00018-015-1952-9

**Published:** 2015-06-17

**Authors:** Kota Saito, Toshiaki Katada

**Affiliations:** Department of Physiological Chemistry, Graduate School of Pharmaceutical Sciences, University of Tokyo, 7-3-1 Hongo, Bunkyo-ku, Tokyo, 113-0033 Japan

**Keywords:** COPII, Collagen, Chylomicron, TANGO1, cTAGE5

## Abstract

Cargo proteins exported from the endoplasmic reticulum to the Golgi apparatus are typically transported in coat protein complex II (COPII)-coated vesicles of 60–90 nm diameter. Several cargo molecules including collagens and chylomicrons form structures that are too large to be accommodated by these vesicles, but their secretion still requires COPII proteins. Here, we first review recent progress on large cargo secretions derived especially from animal models and human diseases, which indicate the importance of COPII proteins. We then discuss the recent isolation of specialized factors that modulate the process of COPII-dependent cargo formation to facilitate the exit of large-sized cargoes from the endoplasmic reticulum. Based on these findings, we propose a model that describes the importance of the GTPase cycle for secretion of oversized cargoes. Next, we summarize reports that describe the structures of COPII proteins and how these results provide insight into the mechanism of assembly of the large cargo carriers. Finally, we discuss what issues remain to be solved in the future.

## The requirement of COPII proteins for collagen export from the ER

**Fig. 1:**
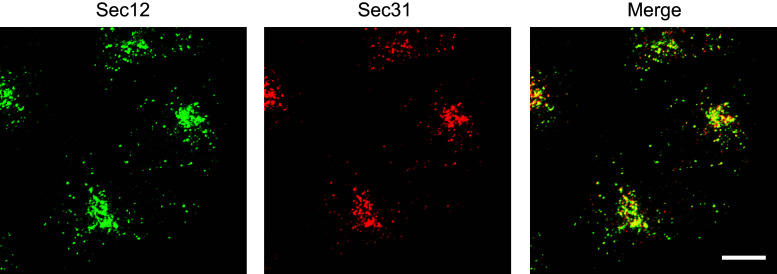
Localization of ER exit sites within HeLa cells stained by antibodies against trans-membrane protein Sec12 (rat monoclonal 6B3) and cytoplasmic Sec31 (BD biosciences mouse monoclonal). HeLa cells were fixed with cold methanol and processed for immunofluorescence staining as described previously [99]. *Bar* 10 μm

Cargoes exiting the endoplasmic reticulum (ER) to the Golgi apparatus are packaged into coat protein complex II (COPII)-coated vesicles that typically have diameters of 60–90 nm [1]. The formation of COPII vesicles occurs in particular regions of the ER called ER exit sites, also known as transitional ER (tER), which stain as punctuated dots scattered throughout the cytoplasm by immunofluorescence analysis of mammalian cells (Fig. 1). The mechanisms to form transport vesicles is highly conserved from yeast to humans. The small GTPase Sar1 is activated by its guanine-nucleotide exchange factor (GEF), Sec12 [2–5]. After activation, Sar1 is recruited to the ER membrane [6–8] and forms the pre-budding complex [9–12], which consists of the inner coat complex Sec23/Sec24 and Sec24-bound cargo molecules [13–15]. Subsequently, the outer coat complex Sec13/Sec31 binds, and this binding event enhances the GTPase-activating protein activity of Sec23, thereby completing coat assembly [16–19]. Sec16 is the other factor essential in COPII biogenesis and functions as a scaffold to interact with particular coat proteins [20–25]. Recently, Sec16 has also been reported to negatively regulate GTP hydrolysis by Sar1 [26–28]. Additional factors involved in vesicle production have been identified, such as p125, TFG-1, and ALG2 [29–38]. The details of conventional COPII biogenesis have been reviewed extensively in other recently published articles [39–46].

Collagens synthesized in the ER fold into hetero- or homo-trimers, which form >300-nm-long rigid structures that are too large to fit into conventional COPII-coated vesicles [47–50]. However, imaging by fluorescent and electron microscopy indicates that collagen I exits the ER via the COPII-dependent process. Stephens and Pepperkok showed that microinjection of a GTPase-deficient mutant of Sar1a (Sar1a H79G) blocks the secretion of collagen I. Moreover, they showed that collagen I exits the ER in structures labeled with Sec24D, but segregates from vesicular stomatitis virus glycoprotein VSVG-ts045, a model of the conventional cargo proteins. The latter result implies that collagen transport to the Golgi is COPII dependent but distinct from conventional cargo trafficking [51]. Mironov et al. strengthened this finding by electron microscopy analysis, which showed that VSVG and collagen I exit the ER by a COPII-dependent process, but from distinct domains. Moreover, it was observed that protrusions from the ER domains in the vicinity of the ER exit sites form carriers containing either VSVG or collagen I. Interestingly, the formation of carriers is COPII dependent but does not seem to involve budding and fusion of COPII-coated vesicles [52].

### Sec23A

The importance of COPII proteins for collagen secretion has also been suggested by analyzing human diseases and animal models (Table 1). Two point mutations in Sec23A genes (F382L and M702V) have been identified as being responsible for cranio-lenticulo-sutural dysplasia (CLSD), an autosomal recessive disorder characterized by late-closing fontanels, facial dysmorphisms, and skeletal defects [53, 54]. Fibroblasts isolated from CLSD patients showed extensive dilation of the ER and accumulation of collagen I within the ER. Both mutations are located close to the binding site of Sec31. Biochemical and structural characterization suggests that Sec23A/F382L cannot recruit Sec13/Sec31 and therefore vesicle budding does not occur [18, 55]. In contrast, Sec23A/M702V is capable of interacting with Sec13/Sec31 and has no appreciable effects on vesicle budding in vitro. Interestingly, the M702V mutation seems to enhance Sar1B GTPase activity through an interaction with Sec13/Sec31 [56]. The zebrafish *crusher* mutation was obtained through a chemical mutagenesis screen to identify genes involved in craniofacial development [57]. Further analysis revealed that *crusher* has a nonsense mutation at residue 402 of the Sec23A gene. *Crusher* chondrocytes have distended ER with accumulated collagen II inside [58], further supporting that Sec23A is required for collagen export from the ER.

**Table 1:** COPII-related proteins reported in human diseases and animal models

Gene	Organism	Diseases or animal models	Phenotype	References
Sar1B	Human	CMRD, Anderson disease, CMRD-MSS	Severe fat malabsorption	[111–113]
	Zebrafish	Morpholino	Lipid absorption deficits	[114]
Sec23A	Human	CLSD	Intracellular accumulation of collagen I, dilation of the ER	[53, 54]
	Zebrafish	Morpholino	Reduced body length, malformation of cranial cartilage	[53]
	Zebrafish	Mutant (*crusher*)	Intracellular accumulation of collagen II, dilation of the ER	[57, 58]
Sec23B	Human	Congential dyserythropoietic anemia type II	Ineffective erythropoiesis	[132, 133]
	Mouse	Knockout	Perinatal lethality	[134]
	Zebrafish	Morpholino	Ineffective erythropoiesis, immature and binucleated erythrocytes	[132]
Sec24A	Mouse	Knockout	Normal development, reduced plasma cholesterol	[135]
Sec24B	Mouse	Mutant	Defects in neural tube closure	[136, 137]
Sec24C	Mouse	Knockout	Embryonic lethality at approximately embryonic day 7	[138]
	Zebrafish	Morpholino	Normal in development, short	[66]
Sec24D	Human	Osteogenesis imperfecta	Disturbed ossification of the skull, craniofacial defects	[69]
	Mouse	Knockout	Embryonic lethality	[70]
	Zebrafish	Mutant (*bulldog*)	Craniofacial defects, defects in collagen II secretion, dilation of ER	[66]
	Medaka	Mutant (*vbi*)	Craniofacial defects, defects in collagen II secretion, dilation of ER	[67]
Sec13	Zebrafish	Morpholino	Defects in craniofacial development, small eyes	[72, 75]
	Zebrafish	Mutant	Hypoplastic digestive organ, small eyes, collagen II accumulation in ER	[74, 76]
Sec31A	Zebrafish	Morpholino	Defects in digestive organ, collagen II accumulation in dilated ER	[74]
TANGO1	Mouse	Knockout	Defects in collagen I, II, III, IV, VII, IX secretion	[82]
Sedlin	Human	SEDT	Short stature, short trunk, degenerative joint, impaired secretion of ECM	[107]

Recently, Sec23A has been identified as a target of the ER-resident transcription factor BBF2H7, also known as Creb3L2 [59]. BBF2H7 is expressed in chondrocytes and normally degraded by the ubiquitin-proteasome pathway, but upon ER stress, the transcription factor is stabilized and transported to the Golgi, then activated by proteolysis with Golgi-localized site-1 protease (S1P) and site-2 protease (S2P). The cleaved N-terminus translocates to the nucleus to upregulate the expression of Sec23A [60–62]. BBF2H7 knockout mice were found to show severe chondrodysplasia. Chondrocytes from knockout mice have expanded ER, where collagen II and cartilage oligomeric matrix protein accumulate in large amounts [59]. A zebrafish mutant carrying a missense mutation in BBF2H7 (*feelgood*) also showed defects in chondrocyte development, and the accumulation of collagen II was observed in distended ER [63]. These results suggest that BBF2H7-mediated transcription activation of Sec23A is necessary for collagen transport in chondrocytes. The requirement of the BBF2H7-Sec23A pathway for collagen transport was also reported for dermal fibroblasts [64].

### Sec24D

The vertebrate possesses four isoforms of Sec24, and these isoforms are considered to fulfill the demands to traffic varieties of cargo molecules, although they appear to be partially redundant in cargo recognition [65]. Recent studies in fish indicated that Sec24D is specifically important for collagen secretion from the ER. Mutagenesis screens performed in medaka and zebrafish independently led to the identification of the nonsense mutations named *vbi* and *bulldog*, respectively [57]. Both mutants showed craniofacial defects, and chondrocytes from these mutants failed to secrete collagen II and displayed dilated ER [66, 67]. Osteogenesis imperfecta, a disorder associated with reduced bone mass, increased bone fragility, and bone deformity, is caused primarily by heterozygotic mutations in genes encoding collagen I (COL1A1 or COL1A2) [68]. A recent clinical study revealed that mutations of Sec24D are also responsible for the osteogenesis imperfecta phenotype. Affected individuals either possess two missense mutations in each Sec24D allele or one missense and the other nonsense mutation. Fibroblasts from patients showed accumulation of collagen I within the dilated ER [69]. Interestingly, knockout of Sec24D in mice revealed early embryonic lethality [70]. These results imply that Sec24D-dependent cargo transport is required for early stages of development, and truncated or mutated forms of Sec24D from fish mutants and patients have marginal activity required for early development, but not sufficient for secreting collagen I from the ER. Interestingly, the expression pattern of Sec24d has been reported to change during development. It is ubiquitously expressed during the early stages of development, whereas the expression is restricted to craniofacial cartilage during later stages of development [66].

### Sec13/31

Sec13 functions by forming individual complexes in different locations within cells. Sec13 is known to constitute the nuclear pore complex (NPC) [71]. However, Sec13 interacts with Sec31 to serve as an outer layer of COPII vesicles. In addition, Sec13 has been recently suggested to form a complex with Sec16, serving as a template for the formation of Sec13/31 outer coats [25].

Townley et al. first reported that zebrafish Sec13 morphants exhibit defects in craniofacial development. In mammalian cells, depletion of Sec13 by siRNA impairs collagen I secretion without affecting conventional cargo transport [72]. Interestingly, a zebrafish mutant originally identified as the small-liver phenotype in a screen was revealed to have a C-terminal truncation of Sec13, which makes it incapable of binding to Sec31 [73]. The mutant fish exhibits a hypoplastic digestive organ and small eyes with disrupted retinal lamination, and chondrocytes of the mutant showed collagen II accumulation in the dilated ER structures [74, 75]. In this context, a Sec31A knockdown by morpholino was performed in fish and showed malformation of the digestive organ, as observed in Sec13 mutants. Moreover, chondrocytes from morphants accumulate collagen II within the dilated ER. These results strongly suggest that defects in digestive organ development and collagen secretion in Sec13 mutant fish are due to the compromised COPII function. The mutation in NPC component Nup107 exhibits failure of retinal lamination, although knockdown of both Sec31A and Sec31B or treatment with brefeldin A, an inhibitor of ER to Golgi trafficking, has no effect on eye development. Thus, the function of Sec13 in the NPC complex appears to be necessary for retinal development [76].

As evidence accumulates, there is no doubt that collagen secretion from the ER requires COPII components. However, these results are not sufficient to conclude whether collagen is directly transported by modified COPII-coated structures, which can accommodate large-sized cargoes, or the COPII requirement for oversized-cargo secretion is limited and indirect. Recently, several molecules associated with ER exit sites have been identified to be specifically required for large cargo secretion and some models have been proposed. We will focus on this topic in the next section.

## Components specifically required for collagen secretion

### TANGO1

**Fig. 2:**
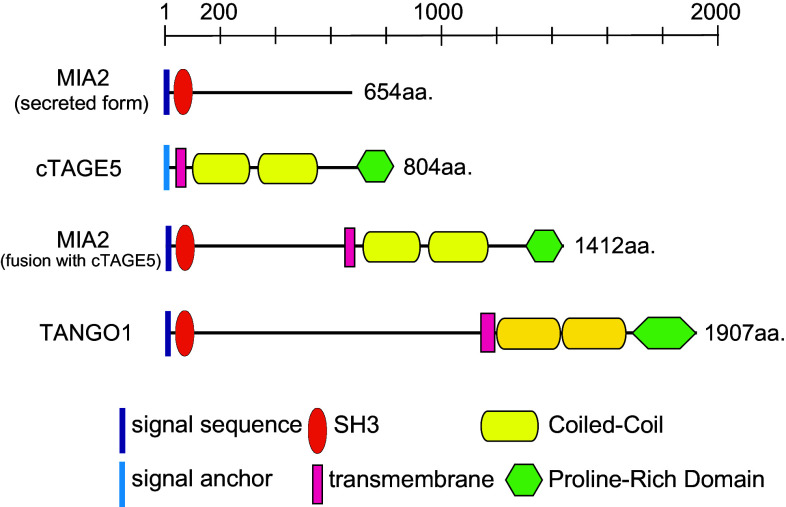
The domain organization of MIA2, cTAGE5, and TANGO1

Genome-wide screening in *Drosophila* S2 cells revealed several genes involved in protein secretion and Golgi morphology [77]. Transport ANd Golgi Organization 1 (TANGO1), also known as Melanoma Inhibitory Activity 3 (MIA3), was isolated in this screening as being involved in the ER to Golgi trafficking. TANGO1, a multi domain-containing protein, with a non-canonical SH3 domain, trans-membrane regions, two coiled-coil domains, and proline-rich domain (PRD), is only conserved through metazoans (Fig. 2). TANGO1 is localized at ER exit sites with the SH3 domain facing into the luminal side and PRD to the cytoplasmic side. Luminal SH3-like folds in MIA-family proteins have unique structural properties when compared with canonical cytoplasmic SH3 domains [78]. PRD of TANGO1 has been shown to interact with Sec23/Sec24, probably in a manner similar to the binding of Sec31 with Sec23/Sec24, because PRD of Sec31 is responsible for the interaction with Sec23/Sec24 [79, 80]. The SH3 domain of TANGO1 is capable of interacting with collagen VII, and a knockdown of TANGO1 impairs collagen VII export from the ER without affecting general transport of proteins. These data suggest that TANGO1 acts as a cargo receptor for collagen VII at ER exit sites [81]. Of note, TANGO1 is not required for collagen I secretion in cultured cells, suggesting that the role of TANGO1 as a cargo receptor is limited to a certain set of molecules and not for all oversized cargoes. In an in vitro vesicle budding assay, TANGO1 was shown to not be exported from the ER along with collagens. In contrast, conventional cargo receptors do exit the ER together with cargo proteins within COPII-coated vesicles. Thus, TANGO1 may employ a unique mechanism for exporting large cargoes from the ER.

TANGO1 knockout mice have been made and exhibit chondrodysplasia, which leads to dwarfing of the fetus, peripheral edema, and neonatal lethality. These phenotypes are probably due to the intracellular accumulation of collagens and the induction of the strong unfolded protein response, especially in the developing skeleton. The TANGO1 knockout was found to show defects in secretion and part of the maturation of collagen I, II, III, IV, VII, and IX from chondrocytes, fibroblasts, endothelial cells, and mural cells [82]. Unlike TANGO1 depletion by siRNA, the TANGO1 knockout inhibited collagen I and VII export from the ER. It is interesting to note that the transcriptional block of the collagen I gene in mice by retrovirus insertion led to embryonic lethality [83], which is a more severe phenotype than the TANGO1 knockout. Whether TANGO1 has direct roles in the secretion and maturation of a broad range of collagens remains to be investigated; however, we propose that the phenotype of knockout animals is an accumulative outcome because of impaired secretion of a limited number of collagens. Of note, it has been reported that TANGO1 in drosophila is also involved in collagen IV secretion from the ER [84, 85].

### cTAGE5

Cutaneous T cell lymphoma-associated antigen 5 (cTAGE5), also known as meningioma-expressed antigen-6 (MGEA6), which was originally isolated as tumor-specific antigens for several types of cancer, is a close homolog to TANGO1 in mammalian cells [86–89]. Although cTAGE5 lacks the N-terminal long luminal stretch when compared with TANGO1, it contained a trans-membrane region, two coiled-coil domains, and a PRD located at the C-terminus, and it localizes to the ER exit sites (Fig. 2). The PRD of cTAGE5 also binds to Sec23/Sec24. cTAGE5 directly interacts with TANGO1 through one of the coiled-coil domains. Cells depleted of cTAGE5 by siRNA accumulate collagen VII within the ER, suggesting that cTAGE5 acts as a co-receptor of TANGO1 at ER exit sites [90].

cTAGE5 is conserved throughout vertebrates and forms a multigene family with nine pseudogenes in humans and possibly in other primates [91]. cTAGE5 expression is fairly ubiquitous, but tissue-specific alternative splicing produces longer forms of cTAGE5, designated as MIA2 (Fig. 2). MIA2 expression is only restricted to hepatocytes, and like TANGO1, it contains an N-terminal SH3-like fold. Mice possessing point mutations in MIA2 have lower circulating VLDL, LDL, HDL, and triglycerides [92]. Further investigation is required to determine whether MIA2 acts as a cargo receptor for oversized cargo. MIA2 is known to have a shorter secreted form, and its role in carcinogenesis has been reported (Fig. 2) [93–97].

**Fig. 3:**
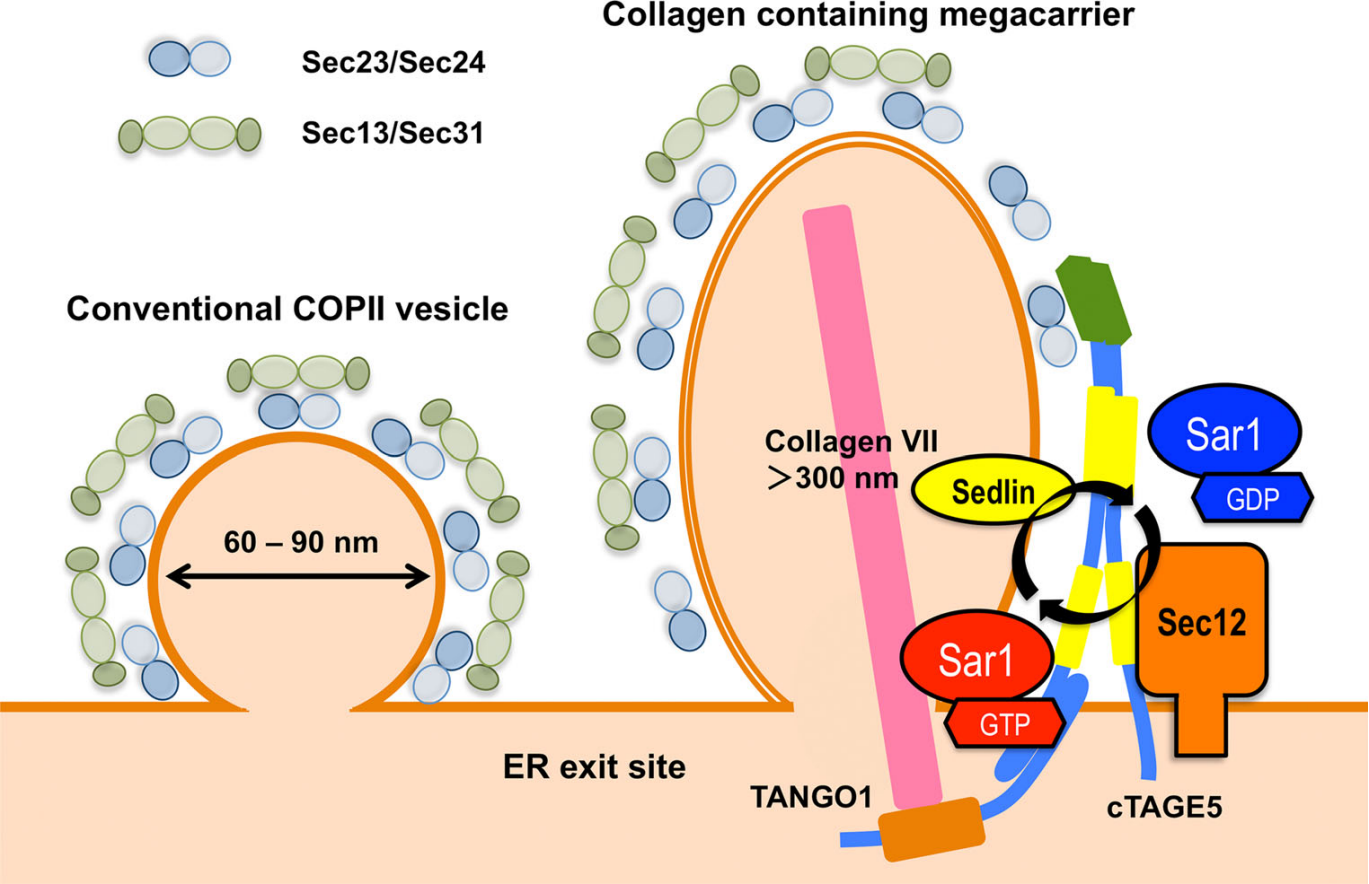
Schematic of conventional COPII-vesicle budding and cTAGE5/TANGO1-mediated collagen export. Collagen secretion may require tight regulation of the Sar1 GTPase cycle. The cTAGE5/Sec12 complex efficiently activates Sar1, whereas TANGO1/Sedlin enhances its hydrolysis for collagen export

Based on the experimental data described above, a model for collagen VII export by the cTAGE5/TANGO1 complex has been proposed (Fig. 3) [81, 90, 98]. The cTAGE5/TANGO1 complex at ER exit sites binds to collagen VII via the luminal SH3-like fold of TANGO1 and Sec23/Sec24 through the PRDs of both cTAGE5 and TANGO1. The interaction of these two PRDs with Sec23/Sec24 inhibits the recruitment of Sec13/Sec31, thereby delaying the Sar1 GTP hydrolysis required for vesicle formation. Once collagen VII is accommodated in a COPII carrier of the right size, collagen VII may dissociate from TANGO1, which would lead to the dissociation of the PRDs from Sec23/Sec24. Sec13/Sec31 may then be recruited and complete the carrier formation. Further experimental validation is required for proving this hypothesis.

### Sec12

Immunoprecipitation following mass spectrometry analysis revealed Sec12 as a new binding partner of cTAGE5 [99]. Sec12, also known as prolactin regulatory element-binding protein in mammalian cells, is a type II transmembrane protein with WD-40 folds and acts as a GEF for Sar1 [5, 100]. Sec12 binds directly to one of the coiled-coil domains of cTAGE5, and this interaction does not exert any changes to the GEF activity of Sec12 toward Sar1. The interaction, however, is necessary for Sec12 to correctly localize to the ER exit sites, as a knockdown of cTAGE5 leads to the dispersion of Sec12 throughout the ER. Interestingly, the cTAGE5 knockdown inhibits collagen VII secretion, but has no effects on general protein secretion, indicating that conventional cargoes can be secreted as long as Sec12 is present within the ER. Thus, Sec12 recruitment to the ER exit sites by interaction with cTAGE5 appears to be specifically required for collagen VII to exit from the ER [99]. As Sec12 is a GEF for Sar1, these data imply that collagen export from the ER requires high levels of activated Sar1 in the vicinity of ER exit sites (Fig. 3).

### Sly1-syntaxin18

Nogueira et al. recently reported that Sly1 interacts with the cytoplasmic domain of TANGO1 in the presence of a crosslinker. Sly1, one of the Sec1/Munc18 (SM) proteins involved in the membrane-fusion reaction, is known to interact with ER-specific target-soluble *N*-ethylmaleimide-sensitive fusion protein-attachment proteins (t-SNAREs), syntaxin17 and syntaxin18 [101, 102]. A knockdown of Sly1 or syntaxin18 specifically blocks secretion of collagen VII, but not collagen I or other conventional cargoes exported from the ER [103]. Recently, a model of collagen VII transport incorporating these findings was reported, suggesting that sly1-syntaxin18-dependent fusion of recycling membranes such as the ER-Golgi intermediate compartment is responsible for enlarging the COPII-mediated carrier, in which formation would be triggered by the action of cTAGE5/TANGO1 [103, 104].

### Cul3-KLHL12

Jin et al. recently reported that ubiquitylation of COPII components is involved in large cargo secretions. Mouse ES cells depleted with ubiquitin ligase CUL3 form tightly packed cell clusters, suggesting the aberrant deposition of the extracellular matrix (ECM). KLHL12 was isolated as an adaptor of CUL3, which binds and shows a similar phenotype to CUL3 when knocked down in ES cells. Interestingly, CUL3-KLHL12 monoubiquitylates Sec31, and this ubiquitylation promotes the formation of enlarged COPII-coated structures (200–500 nm in diameter) where KLHL12 is also present. In addition, the formation of these enlarged COPII structures is required for collagen I and IV transport [105].

### Sedlin

Sedlin, also known as TRAPPC2, is a component of the TRAnsport Protein Particle (TRAPP) complex, which is involved in the tethering of vesicles during ER to Golgi and intra-Golgi transport [106]. Sedlin has been identified as a gene mutated in spondyloepiphyseal dysplasia tarda, an X-linked skeletal disorder characterized by disproportionately short stature with a short trunk and degenerative joints, and chondrocytes from patients show impaired secretion of ECM molecules [107]. Venditti et al. recently showed that Sedlin is localized to the ER exit sites by interaction with TANGO1 and seems to directly interact with the GTP-bound form of Sar1. Knockdown of Sedlin leads to the accumulation of an activated form of Sar1 at ER exit sites and specifically blocks the secretion of collagen I and II from chondrocytes and fibroblasts. The authors suggest that Sedlin regulates the Sar1 GTPase cycle for controlling collagen exit from the ER (Fig. 3) [108].

As described above, collagen secretion from the ER appears to be regulated by specialized factors, which would modify the function of conventional COPII proteins. A cargo receptor complex cTAGE5/TANGO1 is proposed to regulate the Sar1 GTPase cycle by interacting with Sec12, in addition to the binding to Sec23/Sec24 for possible competition with Sec13/Sec31; CUL3-KLHL12 monoubiquitylates Sec31 for enlarging the carriers, and molecules engaged in tethering and fusion, Sedlin and Sly1-syntaxin18, are also involved in the secretion of large proteins.

## Chylomicron secretion

### Sar1B

Chylomicrons synthesized in the enterocyte ER differ in size under different conditions (75–450 nm), but some are considered to be larger than conventional COPII vesicles [109, 110]. Chylomicron retention disease (CMRD), Anderson disease, and CMRD with the neuromuscular disorder Marinesco-Sjogren syndrome (MSS) are all inherited disorders of severe fat malabsorption with impaired chylomicron transport and found to be associated with mutations in Sar1B (Table 1) [111]. The mutations in these diseases mostly lie in the nucleotide binding pockets of Sar1B, indicating the importance of the Sar1 GTPase cycle [111–113]. The zebrafish model of Sar1B deficiency based on a morpholino knockdown showed a similar phenotype, where dietary lipids accumulate in enterocytes of mutant fish [114]. Of note, the fish also exhibit defects in craniofacial cartilage associated with abnormal collagen II secretion, although these defects are not normally seen with CMRD and related diseases.

Interestingly, Sar1B appears to have unique characteristics when compared with the function of Sar1A. Based on the biochemical analysis of a CLSD mutant, Sar1B has been suggested to have a weaker affinity than Sar1A toward Sec13/Sec31. The loose interaction of Sec13/Sec31 with Sar1B may facilitate the formation of a more flexible outer coat that can then accommodate large cargoes [55, 115].

## Structural analysis of COPII proteins that provide insight into the assembly of large cargo carriers

Cryo-electron microscopic analysis of purified Sec23/Sec24 and Sec13/Sec31 showed that these two complexes can co-assemble into the cage-like structure from a cuboctahedron with a diameter of 60 nm to an icosidodecahedron with a diameter of 100 nm [116]. However, these structures are apparently not large enough to accommodate large cargoes such as collagens and chylomicrons. O’Donnell et al. recently reported that Sec13/Sec31 could also form tubules with 330-nm-long hollow cylindrical interiors with a diameter of 30 nm [117].

COPII-dependent tubule formation has also been reported in semi-intact cells, which were treated with an activated form of Sar1 (Sar1 H79G) [7, 118]. In addition, several reports indicate that artificial liposomes can be tubulated by incubation with Sar1 H79G or with Sar1 in the presence of non-hydrolyzable GTP analogs such as GMP-PNP and GTPγS [8, 119–122]. These data imply that secretion of large cargoes requires either large amounts or stabilized GTP-bound Sar1, which is consistent with the proposed function of Sec12 in collagen secretion described above (Fig. 3) [99]. By using cryo-electron tomography and subtomogram averaging, Zanetti et al. showed that giant unilamellar vesicles incubated with Sec12, Sar1, Sec23/Sec24, Sec13/Sec31, and GMP-PNP generate tubules coated with COPII proteins. The arrangement of the inner and outer coats into these tubules is structurally connected to but distinct from that of conventional COPII vesicles reported previously [10, 116, 123–127]. It should be noted that the architecture of assembly of these tubules is quite different from the in vitro-assembled Sec13/31 tubules described above [117].

Although yet to be proven, these tubules may be involved in large cargo transport, and it is interesting to speculate that tubulation is dependent on Sec31 monoubiquitylation. In this regard, a recent study of *S. cerevisiae* showed that the function of Sec13 in COPII vesicle formation may be to rigidify the COPII outer complex for increasing membrane-bending capacity [128]. In addition, phosphorylation of Sec31 by casein kinase II has been reported to reduce its affinity to Sec23, although its involvement in large cargo secretion remains unclear [129]. Future work is expected to reveal whether these modifications of core COPII components are (partly) responsible for the formation of carriers of over-sized cargoes [130, 131].

## Future perspectives

Recent studies on human diseases and animal models have revealed that COPII components are crucial not only for conventional cargo export but also for the export of large proteins and protein complexes from the ER. Moreover, several factors specifically involved in the secretion of huge proteins have been identified and found to modify the process of COPII-dependent vesicle formation to enable them to secrete from the ER. However, several issues still need to be addressed. A major issue that remains to be resolved is the identification of the carriers responsible for large protein transport. The existence of megacarriers or tubules, which could accommodate large cargoes, has been proposed as described in this review, but the exact entities of and the mechanisms to form these containers are not fully understood.

A second issue is the relationship between the specialized factors identified. The cTAGE5/TANGO1 complex and sly1-syntaxin18 axis are reported to be rather specific for collagen VII transport, but not for collagen I. However, CUL3-KLHL12 and Sedlin have been implicated in collagen I transport. Whether they can cooperate to accomplish the large cargo export or they individually apply distinct mechanisms requires further investigation. Although there are still unresolved matters, recent identification of specialized factors has certainly provided us with clues to solve these problems.

## Abbreviations

CMRD: Chylomicron retension disease
COPII: Coat protein complex II
CLSD: Cranio-lenticulo-sutural dysplasia
ECM: Extracellular matrix
ER: Endoplasmic reticulum
GEF: Guanine-nucleotide exchange factor
MIA: Melanoma inhibitory activity
MSS: Marinesco-Sjogren syndrome
NPC: Nuclear pore complex
TANGO: Transport ANd Golgi Organization
TRAPP: TRAnsport Protein Particle
